# Rembrandt van Rijn, Scholar in His Study

**DOI:** 10.3201/eid1203.AC1203

**Published:** 2006-03

**Authors:** Polyxeni Potter

**Affiliations:** * Centers for Disease Control and Prevention, Atlanta, Georgia, USA

**Keywords:** Art and science, emerging infectious diseases, Rembrandt van Rijn, Scholar in His Study

**Figure:**
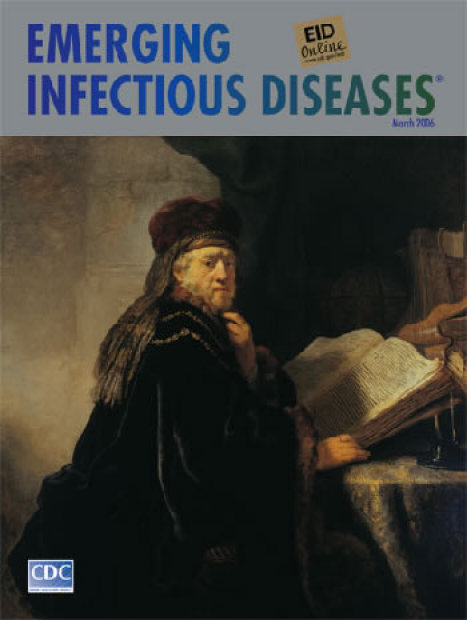
**Rembrandt van Rijn (1606–1669). Scholar in His Study (1634).** Oil on canvas (141 cm × 135 cm). National Gallery in Prague, Czech Republic

For there will be hard data and they will be hard to understand
For the trivial will trap you and the important escape you
For the Committee will be unable to resolve the question
For there will be the arts
and some will call them
soft data
whereas in fact they are the hard data
by which our lives are lived
**John Stone, "Gaudeamus Igitur: A Valediction"**

The "ugly and plebeian face by which he was ill-favored, was accompanied by untidy and dirty clothes, since it was his custom, when working, to wipe his brushes on himself, and to do other things of a similar nature" (*1*), wrote Tuscan biographer Filippo Baldinucci about Rembrandt van Rijn. Though impatient with social conventions and lackadaisical with his own appearance, the artist became known for his impeccable rendition of character and sympathetic view of humanity in his portraits and historical paintings.

Rembrandt was born 400 years ago, during a period of unprecedented prosperity and peace in his native Holland. The son of a miller and a baker's daughter, he was, it was said, "of a different flour" (*2*). The eighth of nine children, he nonetheless received academic encouragement, attended Latin school, and entered the University of Leyden at age 14. Soon he left academic pursuits to become apprenticed to local painter Jacob van Swanenburgh, who worked in the tradition of Hieronymus Bosch (c. 1450–1516), and to study in Amsterdam under renowned history painter Pieter Lastman, who influenced his choice of biblical and mythologic subjects.

The greatest genius of what came to be called Holland's golden age, Rembrandt never traveled abroad. Instead, he absorbed influences indirectly, mostly from the followers of Caravaggio, the Italian master known for the originality of his realism and the use of light and dark (chiaroscuro) to convey intensity and drama (*3*). Rembrandt embraced the technique early, adapted it to his own boldly experimental application of paint and empathetic treatment of the world, and used it throughout his prolific career. Light and shadow became his vehicle for interpreting the human personality, establishing focus, creating mood and feeling, and transporting the viewer beyond the tangible world.

Exuberant self-confidence, native brusqueness, and general aversion to compromise, qualities that lent uniqueness and interest to his art, at times had a deleterious effect on Rembrandt's popularity with art patrons. "After it had become commonly known that whoever wanted to be portrayed by him had to sit to him for some two or three months," wrote Baldinucci, "there were few who came forward" (*4*). Once, while he painted the portrait of a couple with their children, he included in the work the corpse of his monkey, which had just died, to memorialize the pet. "The effect produced by the dead animal so impressed the artist that rather than remove it to satisfy his clients he left the work unfinished" (*4*). At a time when popular taste was turning to classical subjects, Rembrandt drew inspiration from "the humble, the rough, the decayed, the awkward and the heavy" (*5*), whom he painted not as they might have been but just as they were, providing ample clues to their temperament and predicament.

Though he achieved fame and fortune in his lifetime and had many students, among them Gerard Dou, Ferdinand Bol, and Carel Fabritius, the artist came to know personal tragedy. His days were marred with the deaths of his wife and children, disrupted by financial misfortune, and blighted by scandal. A series of self-portraits, more than 60 over 40 years, which trace his artistic and personal growth, show him conflicted, at times a beggar in rags, at times a prosperous man in opulent dress and gold chains. Wealth, respectability, recognition, and following failed to definitively establish his identity, either as esteemed member of society or rebellious outsider.

"I do not care so much for honor as I do for liberty" (*4*), Rembrandt asserted in defiance of his critics. Independence guided all aspects of his life. He was an eclectic art collector, a lover of exotic objects, an unpredictable operator. As his descriptions became less and less detailed and critics thought them sketchlike or unfinished, he retorted that a work is finished "when the master has achieved his intention in it" (*4*). In his later years, entirely oblivious to prevailing technique, he ignored those who thought his color "so heavily loaded that you could lift it from the floor by its nose" (*1*). Rather, his brushstrokes became thicker, his descriptions almost abstract in their reliance on light and dark to convey the "deepest and most lifelike emotion," trademark of his work.

Scholar in His Study, on this month's cover, pays tribute to the pursuit of knowledge, a subject visited often by Rembrandt (A Scholar, The Anatomy Lecture, Philosopher in Meditation, Aristotle Contemplating a Bust of Homer). The rich dark tones are characteristic of the artist's palette as is the interplay of light and shadow providing depth, contrast, and focus. The scholar, placed in the immediate foreground and viewed slightly from below, appears imposing and monumental (*6*). The thick folio lighted against rich brocade, the globe, the books, the surprised, even pained, response to what seems outside interruption imply that the study is of consequence and time of the essence.

Rembrandt's bias is reflected in the respectful rendering of the scholar's appearance. The coat of luxurious velvet embroidered with gold and trimmed with fur, the fantastic head gear, the fine hands suggest elegance, style, status. Despite the symbolism of aging (pallor framed by gray curls, furrowed brow, lusterless eyes inside dark circles, prominent nasal labial lines, fading features) the conjured portrait is of beauty, refinement, dignity, nobility, perseverance, intellectual cultivation. This elder scholar is no old fool.

In the secluded corner where Rembrandt has placed him, the scholar seeks what Aristotle believed all humans naturally desire, knowledge. "Clearly," he wrote in his Metaphysics, "…it is for no extrinsic advantage that we seek this knowledge…since it alone exists for itself" (*7*). In the old volumes stacked in front of him, the scholar searches for the truth about human existence, suffering, danger, hunger, disease, survival, knowing that life slips by before the task is done.

Rembrandt himself searched for the truth in the subjects he painted, in the common people whose complexity he sought to capture. And the penetrating analysis and contemplation characteristic of his self-portraits show no less than compulsion to know himself. His work expanded the world of knowledge, for he did not paint semblance alone. He saw, recognized, and expressed inner values and ideas, universal human traits, natural phenomena; explored, understood, and conveyed emotions; and defined, communicated, and commemorated all these. Piece by piece, in individual paintings and collectively in his life's work, he observed and recorded morsels of truth, seeking to understand and elucidate it.

Whereas his scholar dwelled on words, Rembrandt used color and brushstrokes. For these, along with numbers, notes on the staff, or sheer speculation are the tools for exploring the universe. And so it goes with science and public health. In isolation, like Rembrandt's scholar, in the laboratory, or in the field, public health workers search too, observing, recognizing and meticulously recording relevant information, surveying, delving into the unseen and implied, expanding knowledge.
